# Synthesis and crystal structure of 1,3-bis­(4-hy­droxy­phen­yl)-1*H*-imidazol-3-ium chloride

**DOI:** 10.1107/S2056989019011058

**Published:** 2019-08-16

**Authors:** R. Tyler Mertens, Sean R. Parkin, Samuel G. Awuah

**Affiliations:** a Department of Chemistry, University of Kentucky, Lexington, Kentucky 40506, USA

**Keywords:** crystal structure, imidazolium salt, *N*-heterocyclic carbene, hydrogen bonding, Hirshfeld surface

## Abstract

Imidazolium salts are common building blocks for functional materials and in the synthesis of N-heterocyclic carbene (NHC) as σ-donor ligands for stable metal complexes. The title salt, 1,3-bis­(4-hy­droxy­phen­yl)-1*H*-imidazol-3-ium chloride (IOH·Cl), C_15_H_13_N_2_O_2_^+^·Cl^−^, is a new imidazolium salt with a hy­droxy functionality. The synthesis of IOH·Cl was achieved in high yield *via* a two-step procedure involving a di­aza­butadiene precursor followed by ring closure using tri­methylchloro­silane and paraformaldehyde. The structure of IOH·Cl consists of a central planar imidazolium ring (r.m.s. deviation = 0.0015 Å), with out-of-plane phenolic side arms. The dihedral angles between the 4-hy­droxy­phenyl substituents and the imidazole ring are 55.27 (7) and 48.85 (11)°. In the crystal, O—H⋯Cl hydrogen bonds connect the distal hy­droxy groups and Cl^−^ anions in adjacent asymmetric units, one related by inversion (−*x* + 1, −*y* + 1, −*z* + 1) and one by the *n*-glide (*x* − , −*y* + , *z* − ), with donor–acceptor distances of 2.977 (2) and 3.0130 (18) Å, respectively. The phenolic rings are each π–π stacked with their respective inversion-related [(−*x* + 1, −*y* + 1, −*z* + 1) and (−*x*, −*y* + 1, −*z* + 1)] counterparts, with inter­planar distances of 3.560 (3) and 3.778 (3) Å. The only other noteworthy inter­molecular inter­action is an O⋯O (not hydrogen bonded) close contact of 2.999 (3) Å between crystallographically different hy­droxy O atoms on translationally adjacent mol­ecules (*x* + 1, *y*, *x* + 1).

## Abstract 2

The title compound, 1,3-bis­(4-hy­droxy­phen­yl)-1*H*-imidazol-3-ium chloride (IOH·Cl) is a new imidazolium salt with a hy­droxy functionality.

## Chemical context   

**Figure 1:**
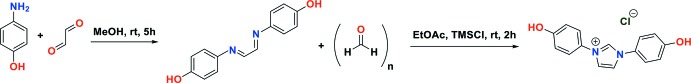
Synthesis of IOH·Cl.

N-Heterocyclic carbenes (NHCs) represent a versatile class of ligand systems for metal-center activation or stabilization in modern organic synthesis (Arduengo *et al.*, 1999 ▸; Benhamou *et al.*, 2011 ▸). Chemically, carbenes are nucleophilic ‘phosphine mimics’ that are high in the order of the Tolman electronic and steric parameter scales, which influences their reactivity. Metal complexes bearing NHC ligands are found in many catalytic reactions (Flanigan *et al.*, 2015 ▸; Hopkinson *et al.*, 2014 ▸; Huynh, 2018 ▸; Marion & Nolan, 2008 ▸; Scholl *et al.*, 1999 ▸; Velazquez & Verpoort, 2012 ▸; Wang *et al.*, 2018 ▸), and recently have shown promise as cytotoxic agents (Garrison & Youngs, 2005 ▸; Lam *et al.*, 2018 ▸; Liu & Gust, 2013 ▸; Mora *et al.*, 2019 ▸; Riener *et al.*, 2014 ▸; Zou *et al.*, 2018 ▸). Imidazolium salts, which are simple salts of the free carbene, are commonly used in many systems in preference to their free carbene counterparts due to their high stability. Unlike the free carbenes, which readily react with water or oxygen (Alder *et al.*, 1995 ▸), imidazolium salts are indefinitely stable. Use of the imidazolium salt does not require Schlenk techniques and the corresponding ‘free’ carbene can be prepared *in situ via* deprotonation with a strong base (*e.g.* NaO^*t*^Bu and NaH) (Arduengo *et al.*, 1991 ▸; McGuinness *et al.*, 2001 ▸; Hauwert *et al.*, 2008 ▸; Voutchkova *et al.*, 2005 ▸). Expanding the functional diversity of NHC ligands will broaden their utility. The synthesis of the novel imidazolium salt in this report offers a unique extension of previously reported imidazolium salts through the addition of phenolic groups, herein referred to as IOH·Cl, for functionalization (see Scheme). The hydroxyl functional group presents the possibility of tethering other chemical groups for varied applications, including catalysis, materials, and biomedicine. The synthesis of IOH·Cl (Fig. 1 ▸) does not require Schlenk techniques and the product is isolated as an air-stable solid that can be stored indefinitely without decomposition. The synthesis is part of a study to develop reaction methods for C—N bond formation from high-oxidation-state transition metals.
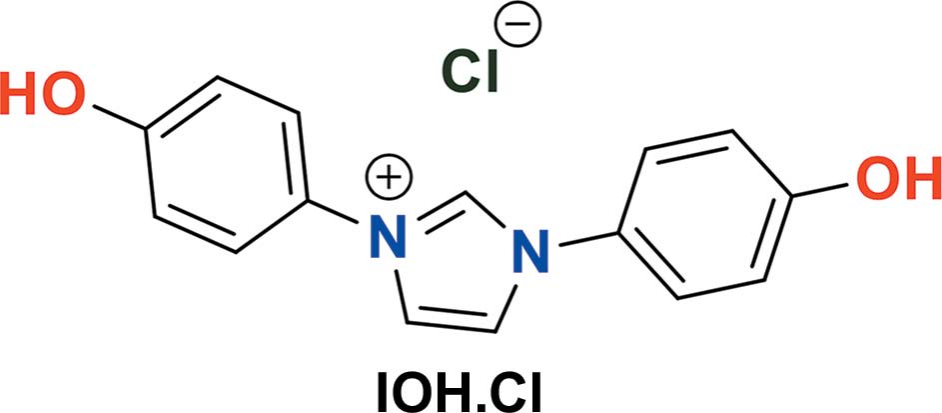


## Structural commentary   

**Figure 2:**
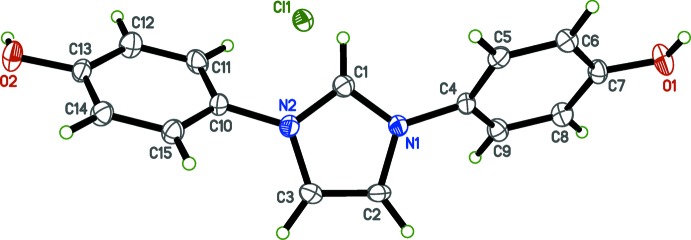
The mol­ecular structure of IOH·Cl, with displacement ellipsoids drawn at the 50% probability level.

In the structure of IOH·Cl (Fig. 2 ▸), there are no unusual bond lengths or angles. The organic cation consists of a central planar imidazolium ring (r.m.s. deviation = 0.0015 Å), with *para*-phenol substituents (C4–C9/O1 and C10–C16/O2) bonded to the imidazolium N atoms [N1—C4 = 1.442 (3) Å and N2—C10 = 1.441 (3) Å]. The phenol groups are out-of-plane, forming dihedral angles with the imidazolium ring of 55.27 (7) and 48.85 (11)° for rings C4–C9 and C10–C15, respectively. The hy­droxy H-atom coordinates were refined freely and are slightly out-of-plane of their respective phenolic groups; the torsion angles are 9.1 (19)° for C6—C7—O1—H1*O* and 11 (2)° for C12—C13—O2—H2*O*.

## Supra­molecular features   

**Figure 3:**
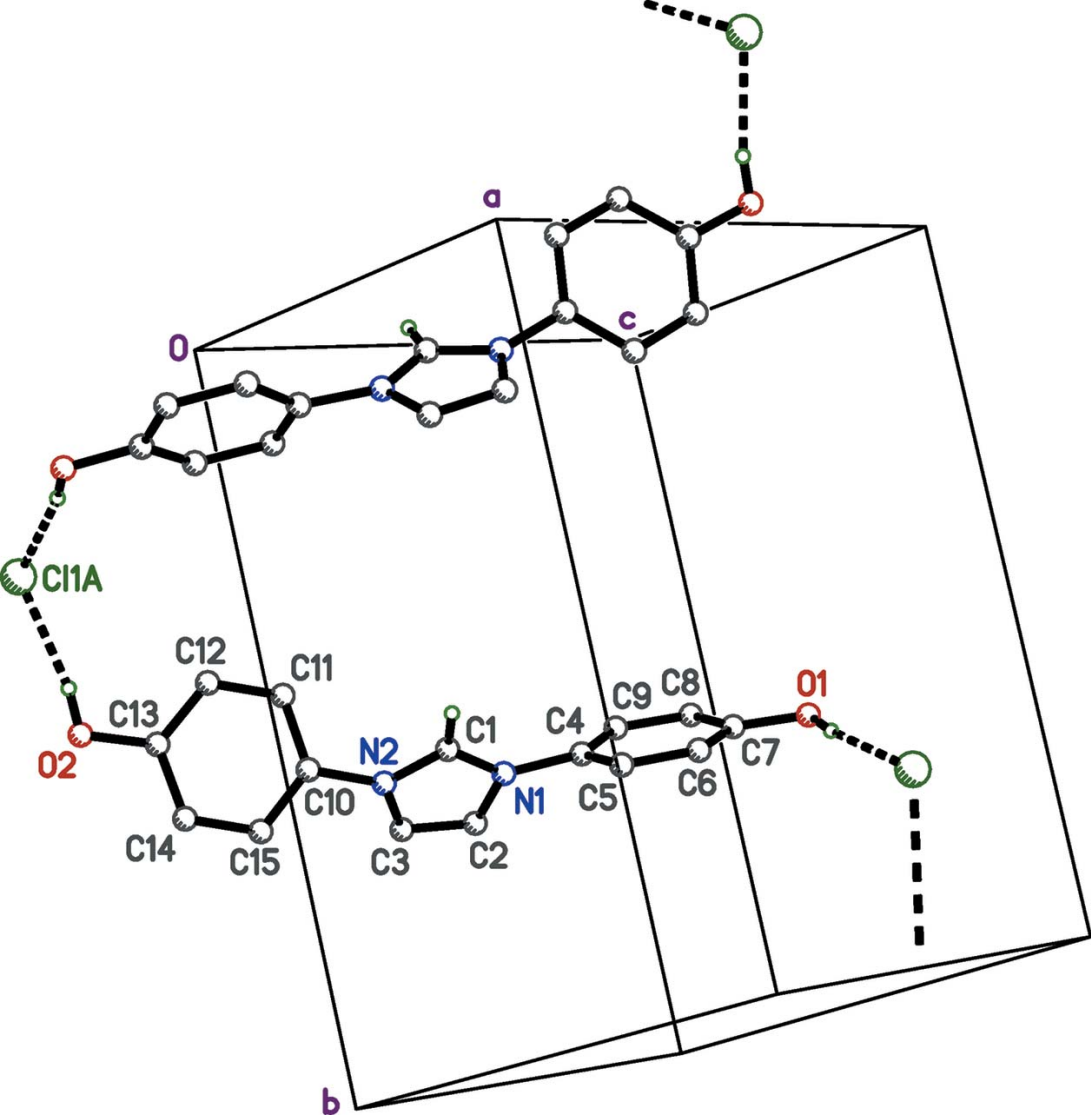
A plot of the O—H⋯Cl hydrogen bonds in crystals of IOH·Cl. These inter­actions, drawn as dashed solid lines, link mol­ecules into head-to-tail zigzag chains that extend parallel to the *b* axis. The unlabelled mol­ecule is related to its labelled counterpart by the crystallographic 2_1_ screw axis (−*x* + 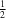, *y* − 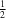, −*z* + 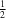).

**Figure 4:**
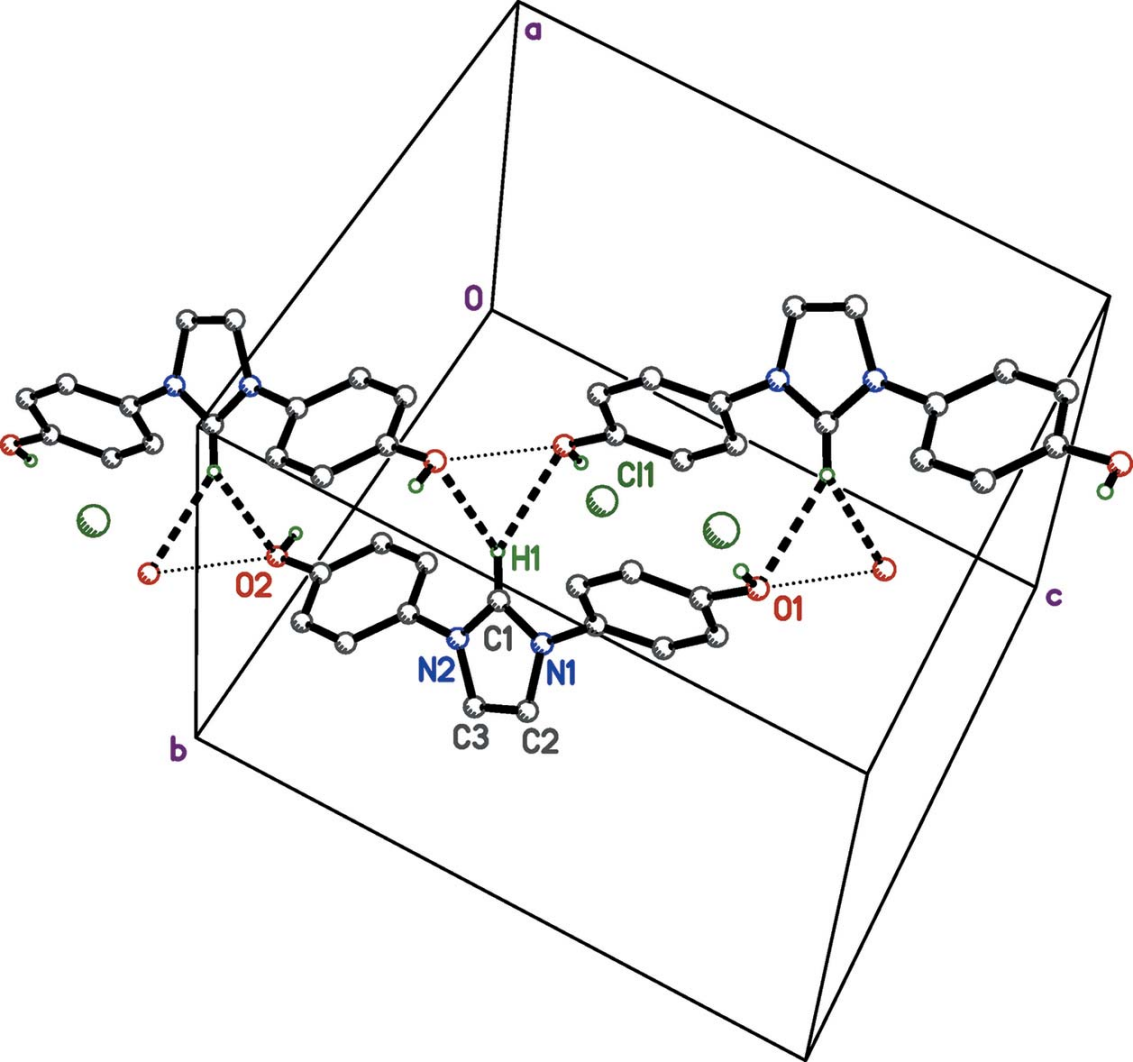
A plot showing the bifurcated C—H⋯O inter­actions (dashed solid lines) and O⋯O close contacts (dotted lines) in crystalline IOH·Cl. The unlabelled mol­ecules are related to the partially labelled mol­ecule by inversion [upper left: (−*x*, −*y* + 1, −*z*); upper right: (−*x* + 1, −*y* + 1, −*z* + 1)].

**Figure 5:**
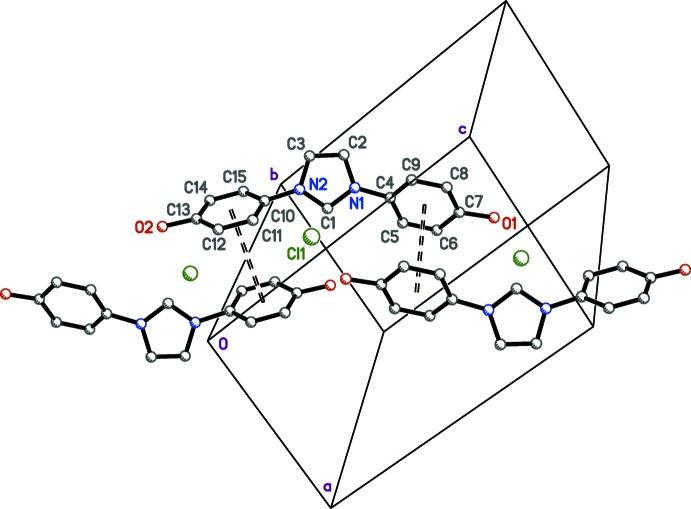
A plot highlighting the π–π stacking (open dashed lines) of benzene rings in crystals of IOH·Cl. Unlabelled mol­ecules are related to the labelled mol­ecule by inversion [lower right: (−*x* + 1, −*y* + 1, −*z* + 1); lower left: (−*x*, −*y* + 1, −*z*)].

**Figure 6:**
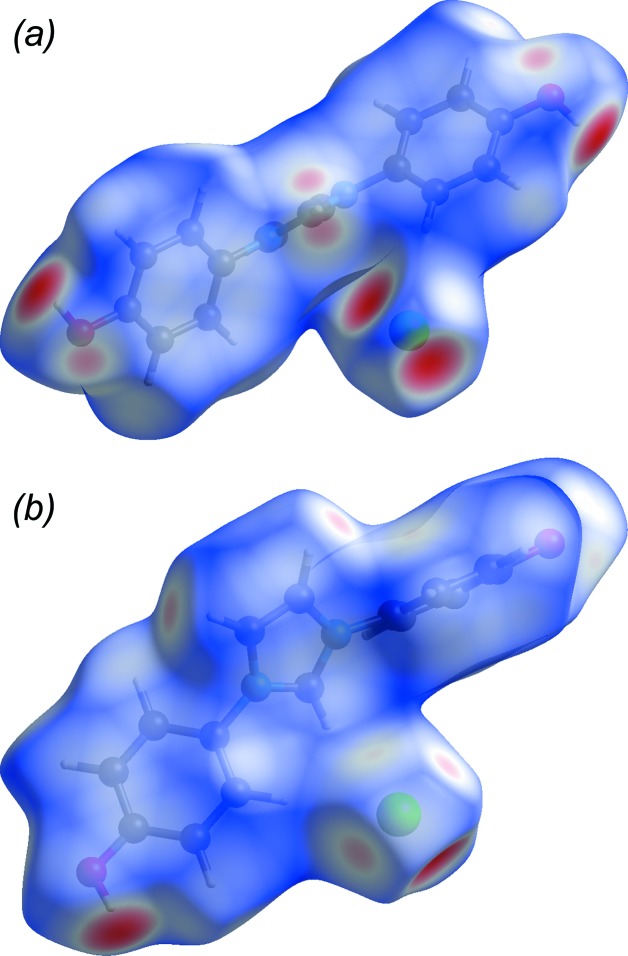
Two views of the normalized contact distances, *d*_norm_, mapped onto the Hirshfeld surface of IOH·Cl. In (*a*), the larger red regions correspond to the O—H⋯Cl hydrogen bonds, while the smaller pink regions correspond to the C—H⋯O bifurcated weak hydrogen bonds. In (*b*), the faint-pink regions in the upper middle of the diagram correspond to close contacts between imidazole-ring C—H groups and Cl^−^ anions.

**Figure 7:**
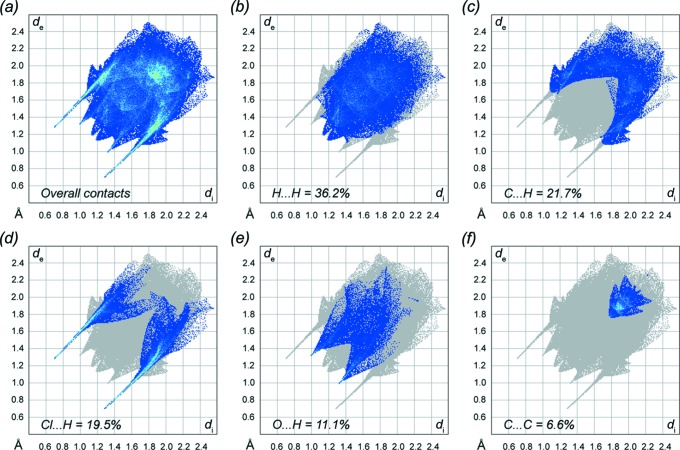
(*a*) The full 2D (two-dimensional) fingerprint plot for IOH·Cl, along with separate plots highlighting the five most important and abundant specific contacts: (*b*) H⋯H, (*c*) C⋯H, (*d*) Cl⋯H, (*e*) O⋯H, and (*f*) C⋯C.

The most prominent inter­molecular inter­actions in the crystals of IOH·Cl are O—H⋯Cl hydrogen bonds. These link the Cl^−^ anion at (*x*, *y*, *z*) to two different IOH·Cl mol­ecules, one related by inversion and the other by the *n*-glide. These hydrogen bonds, *viz.* O1^i^—H1*O* ^i^⋯Cl1 and O2^ii^—H2*O* ^ii^—Cl1 [symmetry codes: (i) −*x* + 1, −*y* + 1, −*z* + 1; (ii) *x* + 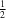, −*y* + 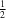, *z* + 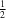; Fig. 3 ▸ and Table 1 ▸], have donor–acceptor distances of 2.975 (2) and 3.012 (2) Å, respectively. Weaker bifurcated C—H⋯O inter­actions occur between imidazole ring atoms (C1—H1) and hy­droxy O atoms (O1^i^ and O2^iii^) on mol­ecules related by different inversion centres. These same hy­droxy O atoms are in close contact with each other, *i.e.* O1^i^⋯O2^iii^ = 2.999 (3) Å [symmetry codes: (i) −*x* + 1, −*y* + 1, −*z* + 1; (iii) −*x*, −*y* + 1, −*z*; Fig. 4 ▸]. In addition to hydrogen bonding, there are offset π–π stacking inter­actions (Fig. 5 ▸). The perpendicular stacking distance between the C4–C9 benzene ring and an inversion-related equivalent at (−*x* + 1, −*y* + 1, −*z* + 1) is 3.560 (3) Å. The overlap of the C10–C15 benzene ring C10–C15 with an inversion-related equivalent at (−*x*, −*y* + 1, −*z*) is weaker, giving a perpendicular stacking distance of 3.777 (3) Å. All hydrogen-bond inter­actions are readily apparent in the Hirshfeld surface and fingerprint plots (McKinnon *et al.*, 2007 ▸; Turner *et al.*, 2017 ▸; Tan *et al.*, 2019 ▸). In Fig. 6 ▸(*a*), the prominent deep-red ellipse-shaped regions represent the O—H⋯Cl hydrogen bonds, while the faint-red regions represent the bifurcated C—H⋯O inter­actions (Table 1 ▸). Short contacts between the imidazole ring and inversion [C2—H2⋯Cl^iv^; symmetry code: (iv) −*x*, −*y* + 1, −*z* + 1] and 2_1_-screw [C3—H3⋯Cl^v^; symmetry code: (v) −*x* − 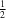, *y* + 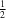, −*z* + 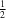] related anions are also apparent (Fig. 6 ▸ *b* and Table 1 ▸). Hirshfeld-surface ‘fingerprint plots’ (Figs. 7 ▸ *a*–*f*) qu­antify the majority of inter­molecular contacts as H⋯H (36.2%; Fig. 7 ▸ *b*) and C⋯H (21.7%; Fig. 7 ▸ *c*). In these diagrams, the O—H⋯Cl hydrogen bonds are indicated by sharp diagonal jutting spikes (Fig. 7 ▸ *d*), while C—H⋯O inter­actions give less-pronounced spikes (Fig. 7 ▸ *e*). C⋯C contacts, which are all as a result of π–π stacking, account for 6.6% of the inter­molecular contacts (Fig. 7 ▸ *f*).

## Database survey   

A search of the Cambridge Structural Database (CSD; Version 5.40, November 2018; Groom *et al.*, 2016) on the three-ring fragment of the title compound yielded over 600 hits, ranging from similar simple salts to metal complexes containing analogous NHC frameworks. A search with H atoms bonded to the three carbons of the imidazole ring gave 180 hits. Of these, 28 had mesityl substituents, including IHOQUS (IMes·Cl; Lorber & Vendier, 2009 ▸) and GAKCAZ (IMes·BF_4_; Bethel *et al.*, 2016 ▸), and 62 had 2,6-diiso­propyl­phenyl groups, including KIDKUG (IPr·ClO_4_; Minaker *et al.*, 2018 ▸), OHURIU (IPr·PF_6_; Rheingold *et al.*, 2015 ▸), TAXLOW (IPr·SiF_5_; Alič *et al.*, 2017 ▸), and XANPEJ (IPr·I; Solovyev *et al.*, 2010 ▸). Structures most similar to IOH·Cl in the present work include the commonly used IMes·Cl (IHOQUS) and IPr·ClO_4_ (KIDKUG), and the unsubstituted phenyl analog IPh·ClO_4_ (DPIMPC; Luger & Ruban, 1975 ▸). A more restrictive search with only *para* substitution allowed on the phenyl rings gave 47 hits, of which 44 were carboxyl­ates that formed extended polymeric structures with metal-containing species. The remaining three, BOGVAV (Wan *et al.*, 2008 ▸), TUPYAF (Garden *et al.*, 2010 ▸), and DAQKOW (Suisse *et al.*, 2005 ▸), have –OMe, –Br, and –OC_12_H_25_ groups at the *para* position. One other structure with comparative functionalization is LEBMUC (Schedler *et al.*, 2012 ▸), which bears bis-meth­oxy groups at the *ortho*-phenyl-ring positions.

## Synthesis and crystallization   

The overall reaction for the synthesis of the title compound is depicted in Fig. 1 ▸. Step 1, *Synthesis of the precursor *N*,*N*′-bis(4-hydroxyphenyl)-1,4-diazabutadiene (1)*: to a round-bottomed flask charged with 15 ml of methanol, 4-phenolaniline (813 mg, 7 mmol) was added and stirred until fully dissolved. Glyoxal (174 mg, 3 mmol) was added to the reaction solution with stirring. Upon addition of glyoxal solution, 40 wt.% in H_2_O, a brown precipitate formed and the solution turned orange. The reaction was further stirred at room temperature for 5 h and the solid was vacuum filtered and washed with cold methanol (612 mg, 85% yield). Step 2, *Synthesis of IOH·Cl*: ethyl acetate (10 ml) was pre-heated to 343 K. To the hot solution was added (1) (200 mg, 1.2 mmol) and paraformaldehyde (36 mg, 1.2 mmol). The reaction mixture was stirred until all of the paraformaldehyde had dissolved. To this was added a solution of tri­methyl­chloro­silane (TMSCl) (0.2 ml, 130 mg, 1.2 mmol) in ethyl acetate (0.15 ml) dropwise over 5 min while stirring. The solution was stirred for 2 h and then placed in a refrigerator (275 K) overnight. The precipitate was collected by vacuum filtration and washed with cold ethyl acetate and ether until the filtrate was colorless, yielding a dark-orange solid (yield 208 mg, 60%). Crystals were grown by slow evaporation of a concentrated solution in acetone.

## Refinement   

Crystal data, data collection, and structure refinement details are given in Table 2 ▸. All H atoms were found in difference Fourier maps. Hy­droxy H-atom coordinates were refined freely, with *U*_iso_(H) = 1.5*U*_eq_(O). Carbon-bound H atoms were included in calculated positions and refined using a standard riding model, with C—H = 0.95 Å and *U*_iso_(H) = 1.2*U*_eq_(C). Refinement progress was checked using an *R*-tensor (Parkin, 2000 ▸), *PLATON* (Spek, 2009 ▸), and *checkCIF* (https://checkcif.iucr.org/).

## Supplementary Material

Crystal structure: contains datablock(s) I, global. DOI: 10.1107/S2056989019011058/su5504sup1.cif

Structure factors: contains datablock(s) I. DOI: 10.1107/S2056989019011058/su5504Isup2.hkl

CCDC reference: 1946122

Additional supporting information:  crystallographic information; 3D view; checkCIF report

## Figures and Tables

**Table 1:** Hydrogen-bond geometry (Å, °)

*D*—H⋯*A*	*D*—H	H⋯*A*	*D*⋯*A*	*D*—H⋯*A*
O1—H1*O*⋯Cl1^i^	0.93 (3)	2.06 (3)	2.975 (2)	169 (3)
O2—H2*O*⋯Cl1^ii^	0.89 (3)	2.13 (3)	3.0118 (19)	171 (3)
C1—H1⋯O1^i^	0.95	2.45	3.280 (3)	145
C1—H1⋯O2^iii^	0.95	2.51	3.271 (3)	137
C2—H2⋯Cl1^iv^	0.95	2.80	3.647 (3)	150
C3—H3⋯Cl1^v^	0.95	2.74	3.655 (3)	163

Symmetry codes: (i) 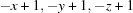; (ii) 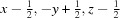; (iii) 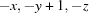; (iv) 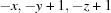; (v) 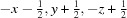.

**Table 2:** Experimental details

Crystal data	
Chemical formula	C_15_H_13_N_2_O_2_^+^·Cl^−^
*M*_r_	288.72
Crystal system, space group	Monoclinic, *P*2_1_/*n*
Temperature (K)	90
*a*, *b*, *c* (Å)	8.1752 (6), 13.2684 (8), 12.7391 (10)
β (°)	100.105 (3)
*V* (Å^3^)	1360.40 (17)
*Z*	4
Radiation type	Mo *K*α
μ (mm^−1^)	0.28
Crystal size (mm)	0.24 × 0.03 × 0.03

Data collection	
Diffractometer	Bruker D8 Venture dual source
Absorption correction	Multi-scan (*SADABS*; Krause *et al.*, 2015 ▸)
*T*_min_, *T*_max_	0.821, 0.928
No. of measured, independent and observed [*I* > 2σ(*I*)] reflections	14925, 3109, 1869
*R*_int_	0.103
(sin θ/λ)_max_ (Å^−1^)	0.649

Refinement	
*R*[*F* ^2^ > 2σ(*F* ^2^)], *wR*(*F* ^2^), *S*	0.049, 0.083, 1.01
No. of reflections	3109
No. of parameters	187
H-atom treatment	H atoms treated by a mixture of independent and constrained refinement
Δρ_max_, Δρ_min_ (e Å^−3^)	0.34, −0.32

Computer programs: *APEX3* (Bruker, 2016 ▸), *SHELXT* (Sheldrick, 2015*a* ▸), *XP* in *SHELXTL* (Sheldrick, 2008 ▸), *SHELXL2018* (Sheldrick, 2015*b* ▸) and *CIFFIX* (Parkin, 2013 ▸).

## supplementary crystallographic information

### Crystal data

**Table:**

C_15_H_13_N_2_O_2_^+^·Cl^−^	*F*(000) = 600
*M**_r_* = 288.72	*D*_x_ = 1.410 Mg m^−^^3^
Monoclinic, *P*2_1_/*n*	Mo *K*α radiation, λ = 0.71073 Å
*a* = 8.1752 (6) Å	Cell parameters from 1847 reflections
*b* = 13.2684 (8) Å	θ = 3.2–25.1°
*c* = 12.7391 (10) Å	µ = 0.28 mm^−^^1^
β = 100.105 (3)°	*T* = 90 K
*V* = 1360.40 (17) Å^3^	Needle, pale yellow
*Z* = 4	0.24 × 0.03 × 0.03 mm

### Data collection

**Table:**

Bruker D8 Venture dual source diffractometer	3109 independent reflections
Radiation source: microsource	1869 reflections with *I* > 2σ(*I*)
Detector resolution: 7.41 pixels mm^-1^	*R*_int_ = 0.103
φ and ω scans	θ_max_ = 27.5°, θ_min_ = 2.2°
Absorption correction: multi-scan (*SADABS*; Krause *et al.*, 2015)	*h* = −10→10
*T*_min_ = 0.821, *T*_max_ = 0.928	*k* = −12→17
14925 measured reflections	*l* = −16→16

### Refinement

**Table:**

Refinement on *F*^2^	Primary atom site location: structure-invariant direct methods
Least-squares matrix: full	Secondary atom site location: difference Fourier map
*R*[*F*^2^ > 2σ(*F*^2^)] = 0.049	Hydrogen site location: mixed
*wR*(*F*^2^) = 0.083	H atoms treated by a mixture of independent and constrained refinement
*S* = 1.01	*w* = 1/[σ^2^(*F*_o_^2^) + 0.9975*P*] where *P* = (*F*_o_^2^ + 2*F*_c_^2^)/3
3109 reflections	(Δ/σ)_max_ < 0.001
187 parameters	Δρ_max_ = 0.34 e Å^−^^3^
0 restraints	Δρ_min_ = −0.32 e Å^−^^3^

### Special details

**Table:**

Experimental. The crystal was mounted using polyisobutene oil on the tip of a fine glass fibre, which was fastened in a copper mounting pin with electrical solder. It was placed directly into the cold gas stream of a liquid-nitrogen based cryostat (Hope, 1994; Parkin & Hope, 1998). Diffraction data were collected with the crystal at 90K, which is standard practice in this laboratory for the majority of flash-cooled crystals.
Geometry. All esds (except the esd in the dihedral angle between two l.s. planes) are estimated using the full covariance matrix. The cell esds are taken into account individually in the estimation of esds in distances, angles and torsion angles; correlations between esds in cell parameters are only used when they are defined by crystal symmetry. An approximate (isotropic) treatment of cell esds is used for estimating esds involving l.s. planes.
Refinement. Refinement progress was checked using *Platon* (Spek, 2009) and by an *R*-tensor (Parkin, 2000). The final model was further checked with the IUCr utility *checkCIF*.

### Fractional atomic coordinates and isotropic or equivalent isotropic displacement parameters (Å^2^)

**Table:**

	*x*	*y*	*z*	*U*_iso_*/*U*_eq_	
Cl1	0.02919 (8)	0.26975 (5)	0.32981 (6)	0.02188 (17)	
N1	0.1115 (3)	0.61456 (15)	0.38285 (18)	0.0172 (5)	
N2	−0.0616 (3)	0.59391 (15)	0.23466 (18)	0.0177 (5)	
O1	0.6632 (2)	0.62630 (14)	0.70648 (16)	0.0267 (5)	
H1O	0.754 (4)	0.658 (2)	0.685 (2)	0.040*	
O2	−0.3960 (2)	0.45255 (13)	−0.15434 (15)	0.0246 (5)	
H2O	−0.407 (3)	0.386 (2)	−0.155 (2)	0.037*	
C1	0.0899 (3)	0.56926 (18)	0.2872 (2)	0.0196 (6)	
H1	0.168035	0.527175	0.261384	0.023*	
C2	−0.0294 (3)	0.66978 (18)	0.3909 (2)	0.0201 (6)	
H2	−0.047100	0.709198	0.450155	0.024*	
C3	−0.1369 (3)	0.65700 (19)	0.2986 (2)	0.0209 (6)	
H3	−0.244440	0.685964	0.280713	0.025*	
C4	0.2593 (3)	0.61151 (18)	0.4634 (2)	0.0170 (6)	
C5	0.4107 (3)	0.64043 (18)	0.4386 (2)	0.0187 (6)	
H5	0.420025	0.657846	0.367563	0.022*	
C6	0.5480 (3)	0.64350 (17)	0.5191 (2)	0.0187 (6)	
H6	0.653195	0.661931	0.503157	0.022*	
C7	0.5329 (3)	0.61976 (18)	0.6233 (2)	0.0195 (6)	
C8	0.3809 (3)	0.58845 (18)	0.6463 (2)	0.0199 (6)	
H8	0.371354	0.570419	0.717145	0.024*	
C9	0.2436 (3)	0.58362 (18)	0.5658 (2)	0.0204 (6)	
H9	0.139637	0.561368	0.580702	0.024*	
C10	−0.1400 (3)	0.55635 (18)	0.1320 (2)	0.0173 (6)	
C11	−0.1393 (3)	0.45368 (19)	0.1120 (2)	0.0222 (7)	
H11	−0.082486	0.408543	0.163846	0.027*	
C12	−0.2225 (3)	0.41836 (19)	0.0158 (2)	0.0231 (7)	
H12	−0.221456	0.348340	0.000365	0.028*	
C13	−0.3082 (3)	0.48456 (19)	−0.0591 (2)	0.0186 (6)	
C14	−0.3043 (3)	0.58696 (19)	−0.0383 (2)	0.0204 (6)	
H14	−0.359489	0.632414	−0.090377	0.025*	
C15	−0.2207 (3)	0.62337 (19)	0.0576 (2)	0.0201 (6)	
H15	−0.218569	0.693606	0.072208	0.024*	

### Atomic displacement parameters (Å^2^)

**Table:**

	*U*^11^	*U*^22^	*U*^33^	*U*^12^	*U*^13^	*U*^23^
Cl1	0.0211 (3)	0.0216 (3)	0.0222 (4)	0.0002 (3)	0.0016 (3)	0.0014 (3)
N1	0.0161 (12)	0.0175 (11)	0.0171 (13)	0.0012 (9)	0.0003 (10)	−0.001 (1)
N2	0.0184 (13)	0.0184 (11)	0.0155 (13)	0.0004 (9)	0.0008 (10)	−0.0011 (9)
O1	0.0184 (11)	0.0370 (12)	0.0223 (12)	−0.0078 (9)	−0.0031 (9)	0.0074 (9)
O2	0.0334 (12)	0.0194 (10)	0.0176 (12)	−0.0022 (9)	−0.0044 (10)	−0.0014 (9)
C1	0.0202 (15)	0.0199 (14)	0.0180 (17)	0.0023 (11)	0.0015 (13)	0.0003 (12)
C2	0.0191 (15)	0.0226 (14)	0.0197 (17)	0.0058 (11)	0.0062 (13)	−0.0025 (12)
C3	0.0180 (15)	0.0222 (14)	0.0222 (17)	0.0054 (12)	0.0028 (13)	−0.0001 (12)
C4	0.0156 (14)	0.0183 (13)	0.0160 (16)	−0.0002 (11)	0.0000 (12)	−0.0012 (12)
C5	0.0222 (16)	0.0182 (14)	0.0158 (16)	0.0015 (11)	0.0035 (13)	−0.0006 (11)
C6	0.0182 (15)	0.0181 (14)	0.0198 (16)	−0.0012 (11)	0.0035 (13)	0.0017 (12)
C7	0.0200 (15)	0.0185 (13)	0.0179 (16)	0.0007 (11)	−0.0021 (13)	0.0016 (12)
C8	0.0228 (16)	0.0238 (15)	0.0135 (16)	−0.0018 (12)	0.0041 (13)	0.0053 (12)
C9	0.0189 (15)	0.0188 (14)	0.0229 (18)	−0.0018 (11)	0.0022 (13)	0.0006 (12)
C10	0.0161 (15)	0.0196 (14)	0.0155 (16)	−0.0014 (11)	0.0005 (12)	−0.0026 (12)
C11	0.0227 (16)	0.0187 (14)	0.0233 (17)	0.0040 (12)	−0.0015 (13)	0.0030 (12)
C12	0.0280 (17)	0.0178 (14)	0.0225 (18)	−0.0012 (12)	0.0015 (14)	0.0001 (12)
C13	0.0182 (15)	0.0238 (14)	0.0138 (16)	−0.0031 (11)	0.0026 (12)	−0.0021 (12)
C14	0.0258 (16)	0.0188 (14)	0.0163 (16)	0.0027 (12)	0.0027 (13)	0.0022 (12)
C15	0.0235 (16)	0.0177 (14)	0.0185 (16)	0.0000 (11)	0.0017 (13)	−0.0010 (12)

### Geometric parameters (Å, º)

**Table:**

N1—C1	1.342 (3)	C5—H5	0.9500
N1—C2	1.384 (3)	C6—C7	1.391 (4)
N1—C4	1.442 (3)	C6—H6	0.9500
N2—C1	1.341 (3)	C7—C8	1.389 (3)
N2—C3	1.385 (3)	C8—C9	1.382 (3)
N2—C10	1.441 (3)	C8—H8	0.9500
O1—C7	1.366 (3)	C9—H9	0.9500
O1—H1O	0.93 (3)	C10—C15	1.380 (3)
O2—C13	1.364 (3)	C10—C11	1.386 (3)
O2—H2O	0.89 (3)	C11—C12	1.376 (4)
C1—H1	0.9500	C11—H11	0.9500
C2—C3	1.350 (4)	C12—C13	1.393 (4)
C2—H2	0.9500	C12—H12	0.9500
C3—H3	0.9500	C13—C14	1.384 (3)
C4—C9	1.383 (4)	C14—C15	1.378 (4)
C4—C5	1.384 (3)	C14—H14	0.9500
C5—C6	1.381 (4)	C15—H15	0.9500
			
C1—N1—C2	109.0 (2)	O1—C7—C6	122.6 (2)
C1—N1—C4	126.4 (2)	C8—C7—C6	120.1 (3)
C2—N1—C4	124.5 (2)	C9—C8—C7	119.8 (3)
C1—N2—C3	108.7 (2)	C9—C8—H8	120.1
C1—N2—C10	126.4 (2)	C7—C8—H8	120.1
C3—N2—C10	124.7 (2)	C8—C9—C4	119.3 (3)
C7—O1—H1O	110.7 (19)	C8—C9—H9	120.3
C13—O2—H2O	111.0 (19)	C4—C9—H9	120.3
N2—C1—N1	107.9 (2)	C15—C10—C11	121.6 (3)
N2—C1—H1	126.1	C15—C10—N2	118.9 (2)
N1—C1—H1	126.1	C11—C10—N2	119.4 (2)
C3—C2—N1	107.0 (2)	C12—C11—C10	118.7 (2)
C3—C2—H2	126.5	C12—C11—H11	120.6
N1—C2—H2	126.5	C10—C11—H11	120.6
C2—C3—N2	107.4 (2)	C11—C12—C13	120.4 (2)
C2—C3—H3	126.3	C11—C12—H12	119.8
N2—C3—H3	126.3	C13—C12—H12	119.8
C9—C4—C5	121.6 (3)	O2—C13—C14	117.9 (2)
C9—C4—N1	118.3 (2)	O2—C13—C12	122.4 (2)
C5—C4—N1	120.1 (2)	C14—C13—C12	119.7 (3)
C6—C5—C4	118.8 (3)	C15—C14—C13	120.4 (2)
C6—C5—H5	120.6	C15—C14—H14	119.8
C4—C5—H5	120.6	C13—C14—H14	119.8
C5—C6—C7	120.4 (3)	C14—C15—C10	119.1 (2)
C5—C6—H6	119.8	C14—C15—H15	120.5
C7—C6—H6	119.8	C10—C15—H15	120.5
O1—C7—C8	117.4 (3)		
			
C3—N2—C1—N1	0.4 (3)	C6—C7—C8—C9	−1.8 (4)
C10—N2—C1—N1	−175.5 (2)	C7—C8—C9—C4	−0.9 (4)
C2—N1—C1—N2	−0.3 (3)	C5—C4—C9—C8	2.6 (4)
C4—N1—C1—N2	−177.5 (2)	N1—C4—C9—C8	−174.5 (2)
C1—N1—C2—C3	0.1 (3)	C1—N2—C10—C15	−135.4 (3)
C4—N1—C2—C3	177.3 (2)	C3—N2—C10—C15	49.3 (4)
N1—C2—C3—N2	0.2 (3)	C1—N2—C10—C11	47.6 (4)
C1—N2—C3—C2	−0.4 (3)	C3—N2—C10—C11	−127.8 (3)
C10—N2—C3—C2	175.7 (2)	C15—C10—C11—C12	−0.5 (4)
C1—N1—C4—C9	−127.9 (3)	N2—C10—C11—C12	176.5 (3)
C2—N1—C4—C9	55.3 (3)	C10—C11—C12—C13	−1.2 (4)
C1—N1—C4—C5	55.0 (4)	C11—C12—C13—O2	−178.2 (3)
C2—N1—C4—C5	−121.8 (3)	C11—C12—C13—C14	2.5 (4)
C9—C4—C5—C6	−1.5 (4)	O2—C13—C14—C15	178.5 (3)
N1—C4—C5—C6	175.5 (2)	C12—C13—C14—C15	−2.2 (4)
C4—C5—C6—C7	−1.2 (4)	C13—C14—C15—C10	0.5 (4)
C5—C6—C7—O1	−176.8 (2)	C11—C10—C15—C14	0.9 (4)
C5—C6—C7—C8	2.8 (4)	N2—C10—C15—C14	−176.1 (2)
O1—C7—C8—C9	177.9 (2)		

### Hydrogen-bond geometry (Å, º)

**Table:**

*D*—H···*A*	*D*—H	H···*A*	*D*···*A*	*D*—H···*A*
O1—H1*O*···Cl1^i^	0.93 (3)	2.06 (3)	2.975 (2)	169 (3)
O2—H2*O*···Cl1^ii^	0.89 (3)	2.13 (3)	3.0118 (19)	171 (3)
C1—H1···O1^i^	0.95	2.45	3.280 (3)	145
C1—H1···O2^iii^	0.95	2.51	3.271 (3)	137
C2—H2···Cl1^iv^	0.95	2.80	3.647 (3)	150
C3—H3···Cl1^v^	0.95	2.74	3.655 (3)	163

Symmetry codes: (i) −*x*+1, −*y*+1, −*z*+1; (ii) *x*−1/2, −*y*+1/2, *z*−1/2; (iii) −*x*, −*y*+1, −*z*; (iv) −*x*, −*y*+1, −*z*+1; (v) −*x*−1/2, *y*+1/2, −*z*+1/2.
